# Draft Genome Sequence of *Streptomyces gancidicus* Strain BKS 13-15

**DOI:** 10.1128/genomeA.00150-13

**Published:** 2013-04-18

**Authors:** Shailesh Kumar, Navjot Kaur, Nitin Kumar Singh, Gajendra Pal Singh Raghava, Shanmugam Mayilraj

**Affiliations:** Microbial Type Culture Collection and Gene Bank; (MTCC), CSIR-Institute of Microbial Technology, Chandigarh, Indiaa;; Bioinformatics Centre, CSIR-Institute of Microbial Technology, Chandigarh, Indiab

## Abstract

We report the 7.3-Mbp genome sequence of *Streptomyces gancidicus* strain BKS 13-15, isolated from mangrove sediment samples collected from the Bhitar Kanika Mangrove Reserve Forest, Odissha, India. The draft genome of strain *Streptomyces gancidicus* strain BKS 13-15 consists of 7,300,479 bp with 72.6% G+C content, 6,631 protein-coding genes, and 71 RNAs.

## GENOME ANNOUNCEMENT

Waksman and Henrici proposed the genus *Streptomyces* in 1943 (1). *Streptomyces* species are well known by a linear chromosome, complex morphological differentiation, and an ability to produce many bioactive secondary metabolites containing important compounds for pharmaceutical and agrochemical uses (2, 3). Suzuki proposed *Streptomyces gancidicus* in 1957 (4). The organism in this study is *Streptomyces gancidicus* strain BKS 13-15, a Gram-positive bacterium isolated from a Bhitar Kanika soil sample from Odissha, India.

The genome of *Streptomyces gancidicus* strain BKS 13-15 was sequenced using the Illumina-HiSeq 1000 paired-end technology, which produced a total of 81,608,084 paired-end reads (insert size of 350 nucleotides [nt]) of 101 nt. We have used NGS QC toolkit v2.3 (5) to filter the data for high-quality (HQ) (cutoff read length for HQ, 70%; cutoff quality score, 20), vector/adaptor-free reads for genome assembly. A total of 75,080,804 high-quality, vector-filtered reads (~1,011× coverage) were used for assembly with SOAPdenovo v1.05 (at a hash length of 79) followed by GapCloser (at a hash length of 17) software (6). The final assembly contains 174 contigs of total size 7,303,225 bp, with an N_50_ contig length of 79.0 kb; the largest contig assembled measures 351.9 kb. Three contaminated contigs of length 457 nt, 864 nt, and 1,425 nt were removed from assembly, and the genome draft comprising 7,300,479 bp with an N_50_ contig length of 70 kbp and largest contig size of 351.9 kbp was submitted to GenBank (NCBI).

This draft genome (i.e., 171 contigs) comprising 7,300,479 bp was annotated with the help of the RAST (Rapid Annotation using Subsystem Technology) (7) server. A total of 6,631 protein coding regions, 3 rRNAs, and 68 tRNAs were predicted.

RAST annotation indicated that strain *Streptomyces coelicolor* strain A3 (score 502), *Streptomyces lividans* strain TK24 (score 459), *Streptomyces avermitilis* strain MA-4680 (score 386), and *Streptomyces viridochromogenes* strain DSM 40736 (score 378) are the closest neighbors of strain BKS 13-15. The annotation available from the RAST server also indicated that the strain BKS 13-15 has the genes for glycolysis and gluconeogenesis, the pentose phosphate pathway, and the tricarboxylic acid (TCA) cycle. The strain BKS 13-25 also has the genes for catalase (EC 1.11.1.6), ferroxidase (EC 1.16.3.1), urease (alpha, beta, and gamma subunit) (EC 3.5.1.5), fructokinase (EC 2.7.1.4), nicotinamidase (EC 3.5.1.19), peroxidase (EC 1.11.1.7), and galactosidase (alpha and beta) (EC 3.2.1.23). Genes for glycine and serine utilization, i.e., 2-amino-3-ketobutyrate coenzyme A ligase (EC 2.3.1.29), aminomethyltransferase (glycine cleavage system T protein) (EC 2.1.2.10), cystathionine beta-synthase (EC 4.2.1.22), cystathionine gamma-lyase (EC 4.4.1.1), glycerate kinase (EC 2.7.1.31), and d-3-phosphoglycerate dehydrogenase (EC 1.1.1.95), are also present in the annotation. Genes for putative polyketide synthase were also found in the annotation. We have mapped all predicted 6,631 protein coding sequences to Kyoto Encyclopedia of Genes and Genomes (KEGG) pathways (8) with the help of the KEGG Automatic Annotation Server (KASS) server (9) and found that 7 proteins mapped on the streptomycin biosynthesis pathway, i.e., glucokinase (EC 2.7.1.2), phosphoglucomutase (EC 5.4.2.2), myoinositol-1-phosphate synthase (EC 5.5.1.4), myoinositol-1(or 4)-monophosphatase (EC 3.1.3.25), myoinositol 2-dehydrogenase (EC 1.1.1.18), dTDP-glucose 4,6-dehydratase (EC 4.2.1.46), and dTDP-4-dehydrorhamnose 3,5-epimerase (EC 5.1.3.13).

### Nucleotide sequence accession numbers. 

This whole-genome shotgun project has been deposited at DDBJ/EMBL/GenBank under the accession number AOHP00000000. The version described in this paper is the first version, AOHP01000000.
